# Fibrin-Plasma Rich in Growth Factors Membrane for the Treatment of a Rabbit Alkali-Burn Lesion

**DOI:** 10.3390/ijms22115564

**Published:** 2021-05-25

**Authors:** Ronald M. Sánchez-Ávila, Natalia Vázquez, Manuel Chacón, Mairobi Persinal-Medina, Agustín Brea-Pastor, Silvia Berisa-Prado, Luis Fernández-Vega-Cueto, Eduardo Anitua, Álvaro Meana, Jesús Merayo-Lloves

**Affiliations:** 1 Instituto Universitario Fernández-Vega, Fundación de Investigación Oftalmológica, Universidad de Oviedo, 33071 Oviedo, Spain; ronald.sanchezavila@gmail.com (R.M.S.-Á.); natalia.vazquez@fio.as (N.V.); m.chacon@fio.as (M.C.); mairobimedina@gmail.com (M.P.-M.); sberisap@gmail.com (S.B.-P.); lfvc@fernandez-vega.com (L.F.-V.-C.); merayo@fio.as (J.M.-L.); 2 Biotechnology Institute (BTI), 01007 Vitoria, Spain; eduardoanitua@eduardoanitua.com; 3 Instituto de Investigación Sanitaria del Principado de Asturias (ISPA), 33071 Oviedo, Spain; 4 Unidad de Bioterio e imagen Preclínica, Universidad de Oviedo, 33071 Oviedo, Spain; vetuniovi@gmail.com; 5 University Institute for Regenerative Medicine and Oral Implantology (UIRMI), 01007 Vitoria, Spain; 6 Centro de Investigación Biomédica en Red en Enfermedades Raras (CIBERER) (U714), ISCII, 28029 Madrid, Spain

**Keywords:** in vitro expansion, limbal stem cell, alkali-burn lesion, fibrin-PRGF membrane, PRP

## Abstract

The purpose of this work is to describe the use of Fibrin-Plasma Rich in Growth Factors (PRGF) membranes for the treatment of a rabbit alkali-burn lesion. For this purpose, an alkali-burn lesion was induced in 15 rabbits. A week later, clinical events were evaluated and rabbits were divided into five treatment groups: rabbits treated with medical treatment, with a fibrin-PRGF membrane cultured with autologous or heterologous rabbit Limbal Epithelial Progenitor Cells (LEPCs), with a fibrin-PRGF membrane in a Simple Limbal Epithelial Transplantation and with a fibrin-PRGF membrane without cultured LEPCs. After 40 days of follow-up, corneas were subjected to histochemical examination and immunostaining against corneal or conjunctival markers. Seven days after alkali-burn lesion, it was observed that rabbits showed opaque cornea, new blood vessels across the limbus penetrating the cornea and epithelial defects. At the end of the follow-up period, an improvement of the clinical parameters analyzed was observed in transplanted rabbits. However, only rabbits transplanted with cultured LEPCs were positive for corneal markers. Otherwise, rabbits in the other three groups showed positive staining against conjunctival markers. In conclusion, fibrin-PRGF membrane improved the chemically induced lesions. Nonetheless, only fibrin-PRGF membranes cultured with rabbit LEPCs were able to restore the corneal surface.

## 1. Introduction

Ocular chemical burns are one of the most devastating situations for the ophthalmologist, which require an immediate and intensive evaluation and treatment [1,2]. Chemical burns to the eye or ocular adnexa are responsible for 11.5–22.1% of ocular injuries [3] and have potentially relevant long-term consequences to the vision and overall quality of life [4,5,6,7]. Most injuries occur in young men, and exposure results from accidents at work, at home, or during an assault [2,8].

Acidic or alkaline agents usually cause ocular chemical burns. Alkaline substances are more damaging to the eye than acidic substances because they have the inherent ability to penetrate the ocular structures due to their hydrophilic and lipophilic properties [5,9]. The deleterious effect of caustication depends on the nature and type of substance involved, as well as the length of time the substance was in contact with the eye [10], being the extent of ocular surface damage one of the most important prognostic factors for the visual outcome [1,2,9,11,12,13].

Several medical treatments and different surgical procedures (amniotic membrane, penetrating keratoplasty, keratoprosthesis) have been used for the treatment of ocular alkali-burns [2,14,15]. Their success rate being conditioned by the phase of the disease: immediate, acute, early repair and late repair [16], but also by the amount of limbal involvement at the time of the injury.

The limbus is the transitional zone between the sclera and the cornea where epithelial corneal stem cells are located [17,18,19,20,21,22]. The limbus is responsible for renewing the corneal epithelium and acts as a barrier between cornea and conjunctiva, preventing the migration of conjunctival epithelium [21,23]. Limbal stem cell depletion or dysfunction, Limbal Stem Cell Deficiency (LSCD), leads to the loss of the corneal regenerative capacity but also allows conjunctival epithelium to invade the corneal surface leading to opacification, vascularization, the appearance of persistent epithelial defects and inflammation [24]. Clinically, patients present pain, photophobia, loss of vision and blindness in advanced cases.

LSCD has a multifactorial etiology with several primary or secondary causes, with severe chemical burns being a common cause of this disease [14,20,25]. The treatment of LSCD depends on the extent of the affected limbal area. In the case of minor limbal involvement, mechanical debridement of the conjunctival epithelium, whether or not associated to the use of an amniotic membrane, can be enough to restore the corneal surface due to remnant cells of the healthy limbal area [26]. However, in the case of extensive limbal area involvement, treatments range from grafting of contralateral, live related, or cadaveric limbal tissue [27,28,29] to transplant cultured cells of different origin: cornea, oral mucosa and mesenchymal stem cells [30,31,32,33,34,35] or grafting of small pieces of limbal explants directly onto the ocular surface [36,37,38,39,40].

Human amniotic and fibrin membranes are commonly used in the latter two procedures as a substrate providing support to the transplanted cells or limbal explants, respectively [41,42]. Alternative carrier materials have been studied for the transplantation of cultured cells, including coated contact lenses [43], compressed collagen [44] and synthetic membranes [45]. However, these materials have inconveniences such as the threat of transmission of animal-derived pathogens, donor tissue-derived infections or high cost [46].

Plasma Rich in Growth Factors (PRGF) is an autologous platelet-rich plasma that contains a greater number of growth factors than the human serum and other platelet-rich plasmas, which has great versatility, allowing the preparation of different therapeutic formulations including eye drops and fibrin clots [47]. In ophthalmology, fibrin-PRGF membranes have shown effective results when employed as adjuvant treatment in human ocular surface repair [48,49] and as a scaffold [50] for culturing and delivering human Limbal Epithelial Progenitor Cells (LEPCs). However, in the last years, several studies have demonstrated controversial results in the effect of platelet-rich plasmas to promote wound healing and tissue regeneration [51,52]. One reason might be the variability in the preparation protocols that results in distinctive formulations and properties [53,54].

This work aims to prove the efficacy of fibrin-PRGF fibrin membranes to treat an alkali-burn lesion in an animal model with different therapeutic approaches. For this purpose, we studied the transplant of in vitro expanded autologous and heterologous LEPCs on fibrin-PRGF membranes, the Simple Limbal Epithelial Transplantation (SLET) technique with a fibrin-PRGF membrane and the role of the fibrin-PRGF membrane without cultured cells as adjuvant therapy.

## 2. Results

### 2.1. Cell Culture

**Figure 1:**
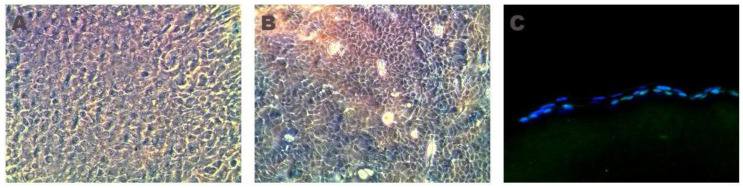
Phase-contrast microscopy of autologous (**A**) or heterologous (**B**) rabbit LEPCs cultured on a fibrin-PRGF membrane displaying their typical polyhedral morphology. Immunofluorescence analysis of fibrin-PRGF membranes cultured with rabbit LEPCs for p63 in green and DAPI in blue (**C**). 100× (phase-contrast microscopy) and 400× (immunofluorescence).

After two days of culture, LEPCs began to migrate from the limbal explants to the culture plate. Cells were flat and round, forming a monolayer immediately adjacent to the explant. After five days, cultures were semiconfluent, and cells were trypsinized and subcultured onto rabbit fibrin-PRGF membranes (groups 2 and 3). These membranes were able to support the cellular growth of rabbit LEPCs while displaying their typical polyhedral morphology. Moreover, immunofluorescence analysis showed that LEPCs expressed a positive stain against the p63 marker (Figure 1).

### 2.2. Rabbit Alkali-Burn Lesion

**Figure 2:**
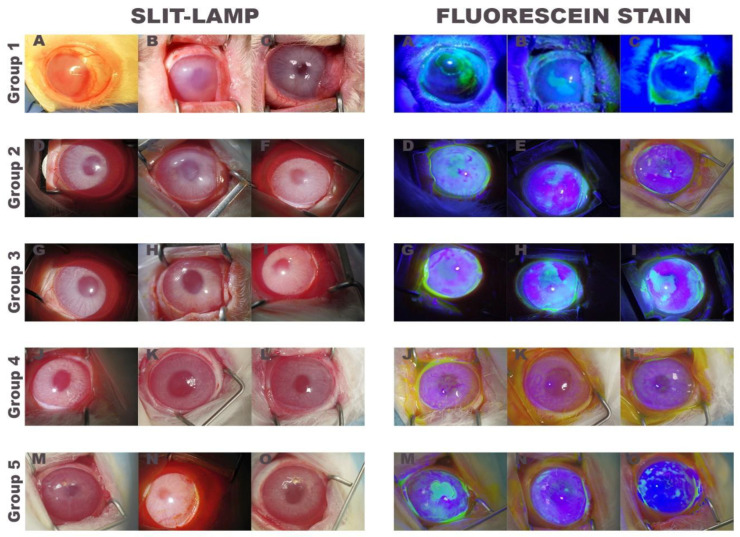
Appearance of rabbit eyes seven days after alkali-burn lesion. Slit-lamp photographs (left) and fluorescein stain photographs (right) of the three treated rabbits of each group. Group 1: only medical treatment (control group), Group 2: fibrin-PRGF membrane cultured with autologous rabbit LEPCs, Group 3: fibrin-PRGF membrane cultured with heterologous rabbit LEPCs, Group 4: fibrin-PRGF membrane SLET and Group 5: fibrin-PRGF membrane without rabbit cultured LEPCs. PRGF: Plasma Rich in Growth Factors; LEPCs: Limbal Epithelial Progenitor Cells; SLET: Simple Limbal Epithelial Transplantation.

Seven days after the alkali-burn lesion, all rabbits showed opaque cornea, new blood vessels across the limbus penetrating the cornea, and epithelial defects shown by fluorescein stain (Figure 2).

Results of clinical evaluation (Table 1) showed that all groups had a mild haze or moderately/severely dense corneal opacity (scores 2–4); with >1/4 of corneal neovascularization (scores 2–4), 2–4 fibrovascular pannus (scores 2 to 4), except one rabbit in the control group (score 1), and epithelial defects affecting up to 75% of the corneal surface (scores 1–3). No significant differences were found between the scores of all groups in the four clinical parameters analyzed.

### 2.3. Clinical Outcome Analysis

**Figure 3:**
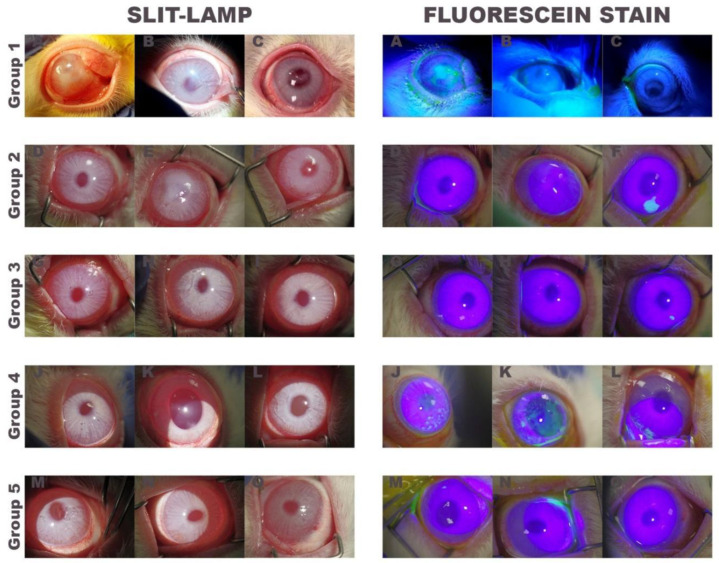
Appearance of rabbit eyes at the end of the follow-up period. Slit-lamp photographs (left) and fluorescein stain photographs (right) of the three treated rabbits of each group. Group 1: only medical treatment (control group), Group 2: fibrin-PRGF membrane cultured with autologous rabbit LEPCs, Group 3: fibrin-PRGF membrane cultured with heterologous rabbit LEPCs, Group 4: fibrin-PRGF membrane SLET and Group 5: fibrin-PRGF membrane without rabbit cultured LEPCs. PRGF: Plasma Rich in Growth Factors; LEPCs: Limbal Epithelial Progenitor Cells; SLET: Simple Limbal Epithelial Transplantation.

All rabbits were evaluated once a week and at the end of the follow-up period (Figure 3).

Results of the clinical evaluation (Table 2) showed that rabbits treated only with medical treatment (control group) retained or worsened the initial clinical diagnosis, with rabbit number one having the most severe degree of corneal opacification, vascularization, and epithelial defects.

In contrast, rabbits in the other treatment groups improved the initial clinical diagnosis. Comparing the final and initial clinical evaluation, this improvement was different between groups. Rabbits transplanted with autologous or heterologous rabbit LEPCs (groups 2 and 3) and rabbits transplanted with fibrin-PRGF membrane SLET (group 4) showed a totally clear cornea (score 0), and only in two cases (in rabbit number two of groups 2 and 4) a mild haze (score 2) was observed. In the case of the rabbits transplanted with fibrin-PRGF membrane without rabbit cultured LEPCs (group 5), all the rabbits showed a haze of minimal density (score 1). Similarly, all transplanted rabbits showed no epithelial defects (score 0) or epithelial defects that affect less than 1/4 of the corneal surface (score 1).

The major differences between groups were observed in the neovascularization and in the number of fibrovascular pannus. In the case of the neovascularization area, the rabbits transplanted with autologous (group 2) or heterologous (group 3) rabbit LEPCs showed an improvement, reducing the affected corneal area by half (from score 3 or 4 to score 1 or 2). In the case of rabbits transplanted with fibrin-PRGF membrane SLET (group 4) and rabbits transplanted with fibrin-PRGF membrane without rabbit cultured LEPCs (group 5), this improvement was not so evident, with one rabbit maintaining the same vascularization (score 3) and two rabbits decreased the vascularized corneal area (score 4 to score 2 or 3). Finally, the improvement in the number of fibrovascular pannus was again most evident in rabbits transplanted with cultured LEPCs (groups 2 and 3), showing a decrease from score 3 or 4 to score 0 or 1. The other groups, rabbits transplanted with fibrin-PRGF membrane SLET (group 4) and rabbits transplanted with fibrin-PRGF membrane without rabbit cultured LEPCs (group 5), also reduced the number of fibrovascular pannus, but to a lesser extent.

One corneal edema was observed in rabbit number two transplanted with fibrin-PRGF membrane cultured with autologous LEPC (group 2).

On the other hand, the percentage of change before and after treatment for each clinical variable measured was compared between the groups (Table 3). Statistically significant differences (*p* < 0.05) were found in corneal neovascularization and fibrovascular pannus, between rabbits transplanted with fibrin-PRGF membrane cultured with autologous (group 2) or heterologous (group 3) rabbit LEPCs, and rabbits treated with only medical treatment (group 1).

### 2.4. Histology and Immunocytochemistry

**Figure 4:**
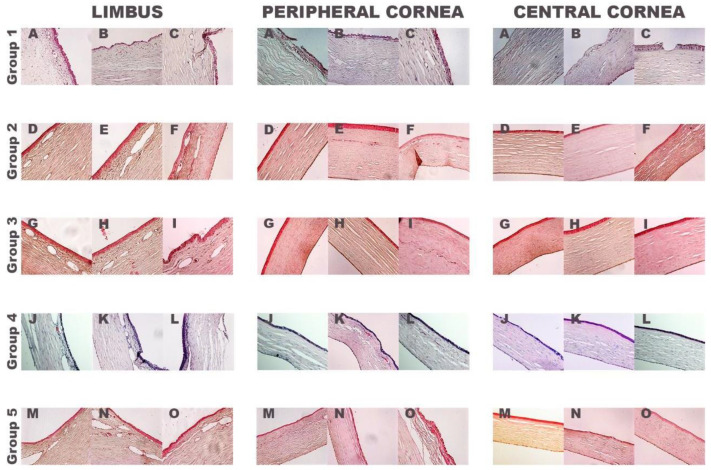
Histological analysis of rabbit corneas. Hematoxylin-eosin photographs of the three treated rabbits’ corneas of each group. Group 1: only medical treatment (control group), Group 2: fibrin-PRGF membrane cultured with autologous rabbit LEPCs, Group 3: fibrin-PRGF membrane cultured with heterologous rabbit LEPCs, Group 4: fibrin-PRGF membrane SLET and Group 5: fibrin-PRGF membrane without rabbit cultured LEPCs. 200×. PRGF: Plasma Rich in Growth Factors; LEPCs: Limbal Epithelial Progenitor Cells; SLET: Simple Limbal Epithelial Transplantation.

Results of the histological evaluation (Figure 4) showed that rabbits treated only with medical treatment (control group) had the limbus area invaded with a conjunctival epithelium characterized by the presence of goblet cells. In the peripheral and central cornea, an abnormal epithelium with a few layers of epithelial cells, ulcers, blood vessels in the underlying stroma and inflammatory cell infiltration were observed in all rabbits in this group.

In contrast, rabbits transplanted with autologous (group 2) or heterologous (group 3) rabbit LEPCs had a normal cornea with a multilayered peripheral and central epithelium with 3–5 layers of epithelial cells, with a slight inflammatory cell infiltration on the matrix and the limbal area showed a normal morphology without the presence of goblet cells. A small peripheral ulcer was observed in rabbit number three of both groups (data are not shown).

However, in the groups of rabbits transplanted with fibrin-PRGF membrane SLET (group 4) and rabbits transplanted with only fibrin-PRGF membrane (group 5), the limbal area included goblet cells penetrating in the peripheral (both groups) and central cornea (rabbit number one of group 4). A small peripheral ulcer was observed in rabbit number three treated with fibrin-PRGF membrane without rabbit cultured LEPC (data are not shown). In the rest of the rabbits, the appearance of the central cornea was normal, in both groups.

**Figure 5:**
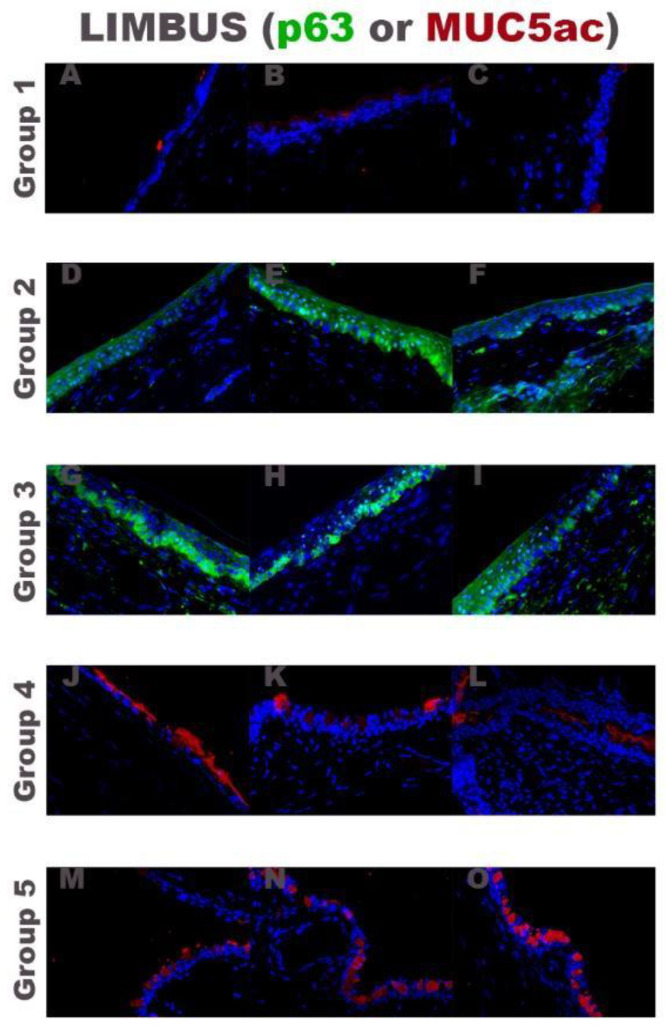
Immunofluorescence analysis of the limbal area with a normal limbal epithelium (p63 in green and DAPI in blue) or with a conjunctival epithelium (MUC5ac in red and DAPI in blue). Group 1: only medical treatment (control group), Group 2: fibrin-PRGF membrane cultured with autologous rabbit LEPCs, Group 3: fibrin-PRGF membrane cultured with heterologous rabbit LEPCs, Group 4: fibrin-PRGF membrane SLET and Group 5: fibrin-PRGF membrane without rabbit cultured LEPCs. 400×. PRGF: Plasma Rich in Growth Factors; LEPCs: Limbal Epithelial Progenitor Cells; SLET: Simple Limbal Epithelial Transplantation.

Immunofluorescence analysis (Figure 5) corroborated the histological analysis showing positive stain against MUC5ac in the limbal area of rabbits treated only with medical treatment (control group), rabbits transplanted with fibrin-PRGF membrane SLET (group 4) and rabbits transplanted with fibrin-PRGF membrane without rabbit cultured LEPCs (group 5). P63 expression was found mainly in the nucleus of the basal layer and a few in the suprabasal layer of the limbal epithelium in the groups of rabbits transplanted with autologous (group 2) or heterologous (group 3) LEPCs.

**Figure 6:**
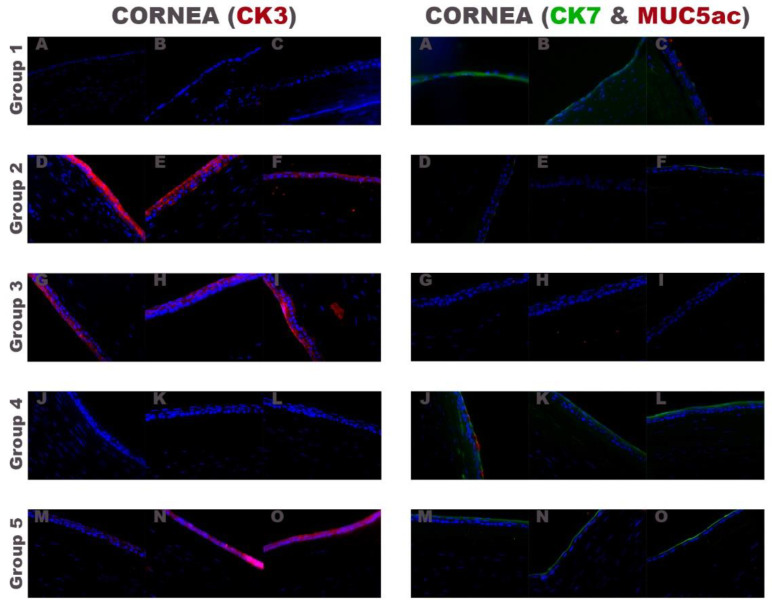
Immunofluorescence analysis of the central cornea with a CK3 positive (in red) multilayered corneal epithelium (left) or a CK7 positive (in green) conjunctival epithelium with or without the presence of MUC5ac positive (in red) cells (right). Group 1: only medical treatment (control group), Group 2: fibrin-PRGF membrane cultured with autologous rabbit LEPCs, Group 3: fibrin-PRGF membrane cultured with heterologous rabbit LEPCs, Group 4: fibrin-PRGF membrane SLET and Group 5: fibrin-PRGF membrane without rabbit cultured LEPCs. 400×. PRGF: Plasma Rich in Growth Factors; LEPCs: Limbal Epithelial Progenitor Cells; SLET: Simple Limbal Epithelial Transplantation.

Moreover, in the groups of rabbits transplanted with fibrin-PRGF membrane cultured with autologous (group 2) or heterologous (group 3) rabbit LEPCs and in the group of rabbits transplanted with fibrin-PRGF membrane without rabbit cultured LEPCs (group 5), CK3 was strongly positive in the intermediate and superficial layers of the central corneal epithelium (Figure 6). In the group of rabbits treated with only medical treatment (control group) and those treated with fibrin-PRGF membrane SLET (group 4), CK7 positive conjunctival epithelium was observed both with (rabbit number three and rabbit number one, respectively) and without MUC5ac positive cells (Figure 6).

## 3. Discussion

Ocular burns account for approximately 15% of eye events [55] and require urgent medical attention, as, if not treated early and properly, the tissues and functioning of the eye may be seriously compromised [56]. After an ocular chemical burn, interventions are aimed at decreasing the extent of the injury, suppressing inflammation, and promoting corneal re-epithelization [14].

The majority of ocular burns do not require surgical interventions, and long-term morbidity rates have been reported as low as 4.5% with medical management alone [57,58,59]. However, an early surgical intervention results in better patient-related outcomes and a lower risk of complications for severe ocular burn-related injury [60].

Surgical treatment of the ocular burns ranges from the application of several membranes to promote epithelization and to reduce inflammation, scarring and neovascularization, in patients with mild to moderate ocular injuries [61,62,63], or the use of membranes as adjuvant therapy, in patients with severe ocular injuries [1,61,64,65,66,67,68,69] to the performance of penetrating keratoplasty [70,71], in patients with extensive stromal scarring, or the use of keratoprosthesis, in patients who have experienced failed previous surgical procedures [72].

In this work, the efficacy of a fibrin-PRGF membrane for the surgical treatment of an alkali-burn lesion was studied in an animal model. Fibrin-PRGF membrane was used as a culture substrate because of its ability to reduce inflammation, vascularization and to stimulate the adhesion and proliferation of LEPCs [49,50,73].

The first step in the treatment of the ocular chemical injury was to irrigate the ocular surface to remove the NaOH. After that, medical treatment with antibiotics, analgesics and anti-inflammatories was administrated to prevent the infections and to reduce the inflammation during the acute phase (7 days). Several classification systems have been described to predict the prognosis of the lesion by grading the severity of the injury [74,75,76]. Since a spectrum of clinical manifestations can be described following chemical injury [5], four parameters were assessed in this study: epithelial integrity, corneal neovascularization, number of fibrovascular pannus and corneal opacity [77,78].

Clinical evaluation of the rabbits showed that all of them had moderately or severely dense corneal opacity, total corneal neovascularization, fibrovascular pannus and epithelial defects seven days after the chemical lesion. A clinical diagnostic with an unfavorable prognosis, since it depends on the extent of corneal, limbal and conjunctival involvement [4,5,7,12]. There were no statistical differences in the scores of the different groups of treated rabbits, indicating that a similar chemical injury was performed.

In this study, the fibrin-PRGF membrane was used with different therapeutic approaches: as a graft that provides a basement membrane for epithelization (group 5), as a membrane that supports the expansion of the autologous (group 2) or heterologous (group 3) rabbit LEPCs to maximize the probability of generating a normal corneal epithelial surface, and in the SLET technique, a simple surgical technique that avoids the consideration of advanced therapy (group 4). These treatments were possible since the fibrin-PRGF membrane has demonstrated to be easily manageable in different types of surgery [48,49] and to support the growth of LEPCs [50].

Rabbit LEPCs grown on fibrin-PRGF membranes were positive for the p63 marker, which has been used to determine the stem cell property of LEPCs [79] being beneficial for long-term graft survival since those cells can proliferate after transplantation.

During post-surgery healing, corneal transparency gradually improved. Within 40 days after transplantation, ocular surfaces were utterly reconstructed with clarity and smoothness, under slit-lamp microscopy, compared to those of normal cornea in the group of rabbits treated with fibrin-PRGF membrane cultured with autologous (group 2) or heterologous (group 3) rabbit LEPCs and in the group of rabbits treated with fibrin-PRGF membrane without cultured LEPCs (group 5). In the groups of rabbits treated only with medical treatment (control group) and rabbits transplanted with fibrin-PRGF membrane SLET (group 4), epithelial defects were detected by slit-lamp examination. One rabbit in the group of fibrin-PRGF membrane cultured with autologous LEPCs showed a corneal edema, which could be explained by the absence of corneal endothelium in a cornea area (data are not shown).

Following clinical observations, the cornea was covered by stratified epithelium displaying corneal phenotype (positive CK3), and the limbal epithelium resembled a normal appearance with limbal stem cells identified by p63 positive labeling in the groups of rabbits treated with fibrin-PRGF membrane cultured with autologous (group 2) or heterologous (group 3) rabbit LEPCs. In contrast, in the other three groups (groups 1, 4, and 5), goblet cells were observed in the limbal epithelium, indicating conjunctivalization into the limbal area. These results seem to indicate that the fibrin-PRGF membrane has a beneficial effect after alkali-burn injury, but it is not able to restore the correct function of a normal cornea by itself [80,81].

In accordance with other studies [82,83], our results showed that a heterologous limbal transplant was as effective as an autologous limbal transplant in treating the alkali-burn lesion. Restoring the ocular surface using expanded heterologous LEPCs represents an encouraging milestone since it would allow the treatment of alkali-burn injuries without compromising the contralateral healthy eye.

Another therapeutic approach, such as the SLET technique, is an even more promising treatment option since it is an easy procedure that does not require in vitro cell culture techniques. Previous studies have shown satisfactory results using this technique [38,40,84,85]. However, in this study, the results obtained have not been satisfactory. These results could be explained because the tarsorrhaphy was opened seven days after the treatment, possibly an inadequate time for integrating the explant onto the ocular surface and to establish a complete corneal epithelium. Better clinical results could have been obtained with a different experimental design.

In the last years, several in vivo studies and clinical trials have demonstrated that fibrin membranes are a suitable scaffold for the culture and transplant of LEPCs, leading in 2015 to the approval of Holoclar^®^ by the European Medicines Agency (EMA), the first Advanced Therapy Medicinal Product (ATMP) containing limbal stem cells in a fibrin scaffold for the treatment of a moderate or severe chemical-induced LSCD [86]. On the other hand, several effective xeno-free techniques of limbal cultivation have been reported to treat chemical ocular lesions [87,88]. However, Holoclar^®^ therapy includes the use of feeder mouse cells and bovine serum, so it is contraindicated for patients with a hyper sensibility to xenogeneic proteins [46], and in most of the xeno-free techniques, an amniotic membrane is used to expand LEPCs, which has some remarkable disadvantages due to the allogeneic origin, the potential pathogen transmission, and high manufacturing costs [89].

In this study, the use of feeder mouse cells was avoided, and limbal explants were used for the expansion of rabbit LEPCs in a simple technique of cellular culture. Although a complete medium with SBF, cholera toxin, among other supplements were used for the expansion of rabbit LEPCs, which was the major limitation of this study, this fact could be modified in the translation of our results to the clinical practice, since human LEPCs have been satisfactorily cultured on a fibrin-PRGF membrane using PRGF as the unique supplement of the culture medium [50]. Thus, a therapy could be developed in which PRGF obtained from patient’s blood could be used for the culture of LEPCs on a fibrin-PRGF membrane for the treatment of patients with severe chemical injuries or a fibrin-PRGF membrane could also be suitable in cases of less severe chemical ocular lesion.

## 4. Materials and Methods

### 4.1. Experimental Design

Fifteen healthy male New Zealand white rabbits (2 months of age and body weight of 2.0–2.5 kg) obtained from the Animal Housing Facility of the University of Oviedo (Oviedo, Asturias, Spain) were used in this work. All animals were treated in accordance with the ARVO Statement to use animals in ophthalmic and vision research and EU Directive 2010/63/EU for animal experiment. The protocols were approved (PROAE 33/2016) by the Committee on the Ethics of Animal Experiments of the University of Oviedo and the Animal Production and Health Service of Asturias. Rabbits were kept under a 12/12 day/night light cycle with food and water ad libitum and were monitored daily.

An alkali-burn lesion was induced in all rabbits, and a week later, all rabbits were clinically examined and treated. Animals were randomly divided into five groups of three rabbits each: rabbits treated only with medical treatment (group 1: control group), rabbits treated with a fibrin-PRGF membrane cultured with autologous (group 2) or heterologous (group 3) rabbit LEPCs, rabbits treated with fibrin-PRGF membrane SLET (group 4) and rabbits treated with a fibrin-PRGF membrane without rabbit cultured LEPCs (group 5). Once a week, rabbits were evaluated (epithelial integrity, corneal neovascularization, fibrovascular pannus and corneal opacity), and after 40 days of clinical follow-up, all rabbits were euthanized, and excised corneas were subjected to histochemical examination.

### 4.2. Cell Culture

Small limbal biopsies (1 × 2 mm) were excised from the limbus of the rabbits’ right eye before the performance of alkali-burn lesion (groups 2 and 3). Limbal biopsies were stored in Leibovitz’s (Thermo Fisher Scientific, Waltham, MA, USA) supplemented with 100 U/mL penicillin and 0.1 mg/mL streptomycin (Sigma-Aldrich, Saint Louis, MO, USA) until culture.

For culture procedure, limbal biopsies were cut into small pieces and cultured for five days on a 12-well culture plate in a 2:1 mixture of Dulbecco’s modified Eagle’s medium (DMEM, Thermo Fisher Scientific) and Ham’s F12 (Thermo Fisher Scientific), supplemented with 5 µg/mL insulin, 8.33 ng/mL cholera toxin, 24 µg/mL adenine, 1.3 ng/mL triiodothyronine, 0.4 µg/mL hydrocortisone, 10 *v*/*v*% fetal bovine serum (FBS) and 100 U/mL penicillin, 0.1 mg/mL streptomycin (Sigma-Aldrich). After five days of culture, rabbit LEPCs were trypsinized, counted in a hemocytometer and subcultured on a fibrin-PRGF membrane for 48 h (groups 2 and 3).

Rabbit fibrin-PRGF membranes were obtained from rabbit blood samples and were processed according to the methodology described by Anitua et al. [90]. Briefly, for each membrane, 5 mL of recovered plasma were incubated in the presence of 250 µL of 10% CaCl_2_ in a 35 mm diameter dish at 37 °C for 30 min. Once a gel was formed, fibrin-PRGF membranes were obtained by flattening for 30 s using a 500 µm fibrin membrane shaper (BTI, Vitoria, País Vasco, Spain).

All cultures were maintained in a humidified incubator at 37 °C with 5% CO_2_, and the medium was changed every two days.

Cellular growth was assessed using a Leica DMIL LED phase-contrast microscope (Leica, Wetzlar, Hesse, Germany), and photos were taken with an attached EC3 camera (Leica). Moreover, fibrin-PRGF membranes cultured with rabbit LEPCs were fixed in ice-cold methanol (Sigma-Aldrich) for 10 min for histological analysis.

### 4.3. Rabbit Alkali-Burn Model

New Zealand white rabbits were treated with Bupaq^®^ (0.01–0.05 mg/kg buprenorphine, Richter Pharma, Wells, Austria) and Metacam^®^ (0.3 mg/kg meloxicam, Boehringer Ingelheim, Ingelheim am Rhein, Germany), and then were intubated and ventilated with isoflurane 2% (Ecuphar Veterinaria, Barcelona, Cataluña, Spain). After topical administration of double anesthetic Colicursi^®^ (0.1% tetracaine and 0.4% oxybuprocaine, Alcon, Geneva, Switzerland), the alkali-burn lesion was performed by applying a gauze soaked in 1 M NaOH (VWR, Radnor, Pennsylvania, USA) on the right eye (left eye served as healthy control) for 10 s. After that, eyes were rinsed with balanced salt solution (Alcon); the central epithelium was removed with eye spears and Tobrex^®^ (3 mg/mL tobramycin, Alcon) and Cetraflux^®^ (3 mg/mL ciprofloxacin, Salvat, Barcelona, cataluña, Spain) eye drops were administered before performing a tarsorrhaphy with 4–0 silk suture (Ethicon, Bridgewater, NJ, USA). Once a day, rabbits received subdermal injections of Metacam^®^, Bupaq^®^ and Alsir^®^ (5 mg/kg enrofloxacin, Esteve, Barcelona, Cataluña, Spain) until treatment.

### 4.4. Clinical Evaluation and Surgical Procedures

Seven days after the alkali-burn lesion, the ocular surface was photographed and evaluated by slit-lamp (Kowa, Dusseldorf, North Rhine-Westphalia, Germany), and the extent of epithelial damage was assessed by fluorescein stain. Opacification, neovascularization, number of fibrovascular pannus and epithelial defects observed in the photographs were evaluated according to the criteria shown in Table 4 [77,78].

Once the rabbits were evaluated, they were anesthetized according to the procedure described above, and the different treatments were carried out. For this purpose, intracorneal vessels were removed, and a fibrin-PRGF membrane was sutured with autologous (group 2), heterologous (group 3) or without rabbit cultured LEPCs (group 5) with cells facing the cornea. In the case of rabbits transplanted with fibrin-PRGF membrane SLET (group 4), a 2 mm limbal biopsy of the contralateral eye was taken, cut into 10 pieces, and glued with Tisseel^®^ (Baxter, Glenview, IL, USA) over a fibrin-PRGF membrane. Then, a second fibrin-PRGF membrane was sutured over the explants with 8–0 absorbable suture (Vicryl^®^, Ethicon).

All rabbits received Vigamox^®^ (5 mg/mL moxifloxacin, Alcon) and Dexafree^®^ (1 mg/mL dexamethasone phosphate, Théa, Barcelona, Cataluña, Spain) eye drops and one subconjunctival injection of Celestone^®^ (3 mg/mL betamethasone, Schering-Plough, Kenilworth, NJ, USA) before performing a tarsorrhaphy with 4–0 silk suture. Once a day, rabbits received subdermal injections of Metacam^®^, Bupaq^®^ and Alsir^®^ 5% for seven days. Tarsorrhaphy was opened after seven days, and rabbits were treated with Vigamox^®^, and Dexafree^®^ eye drops twice a day during the follow-up period. The rabbits in group 1 (control group) received only medical treatment.

### 4.5. Clinical Outcome Analysis

Rabbits’ eyes were monitored with slit-lamp examination, fluorescein staining and photography once a week during the 40 days of the follow-up period. Epithelial integrity, corneal neovascularization, fibrovascular pannus and corneal opacity were evaluated and scored by the same examiner in a masked fashion following the previously described criteria (Table 4).

### 4.6. Histology and Immunocytochemistry

After the follow-up period, rabbits were euthanized by an intravenous overdose of pentobarbital sodium (Vetoquinol, Madrid, Spain) and corneas were excised, rinsed in PBS solution, and fixed in ice-cold methanol for 24 h for histological analysis. Fixed fibrin-PRGF membranes cultured with rabbit LEPCs and rabbit corneas were embedded in paraffin (Thermo Fisher Scientific), and 3 µm sections were cut with a microtome (Leica), placed on commercially treated slides (Thermo Fisher Scientific) and stained with hematoxylin-eosin (Sigma-Aldrich). Immunostaining of the corneal sections using antibody against cytokeratin 3 (CK 3) (1:100; Catalog number: ab68260, Abcam, Cambridge, UK) was used to identify differentiated corneal epithelial cells and antibodies against MUC5ac (1:100; Catalog number: ab212636, Abcam) and cytokeratin 7 (CK 7) (1:100; Catalog number: ab181598, Abcam) were used to identify goblet cells and conjunctival epithelial cells, respectively. Moreover, the antibody against p63 (1:100; Catalog number: ab124762, Abcam) was used to identify progenitor limbal stem cells in fibrin-PRGF membranes cultured with rabbit LEPCs and rabbit corneas. Briefly, samples were deparaffinized, hydrated and rinsed with PBS solution twice for 10 min and permeabilized in a PBS solution containing 0.3% Triton X-100 (VWR, Radnor, PA, USA) for another 10 min. Next, the samples were incubated with primary antibodies containing 10% normal goat serum (Catalog number: ab7481, Abcam, Cambridge, UK) at 4 °C overnight. Subsequently, the samples were incubated with the corresponding secondary antibody (1:500; Catalog number: A-11032 and A-11034, Thermo Fisher Scientific) for 2 h at room temperature. Between incubations, samples were washed three times with PBS for 10 min. Immunolabeled cells were stained with 4′, 6-diamidino-2-phenylindole (DAPI) to allow nuclei visualization. All the samples were examined in a Leica DM6000B fluorescence microscope (Leica), and photographs were taken in the central cornea, peripheral cornea and limbus, identified as the first area where stromal vascular vessels were observed.

### 4.7. Statistical Analysis

Statistical analysis was performed using IBM SPSS Statistics v.22 software (IBM, Armonk, NY, USA). The mean, standard deviation and percentages were used for descriptive analysis. The Wilcoxon test was used to compare the clinical evaluations between the beginning and end of the follow-up period and the Kruskal–Wallis test to compare the results between the groups. A level of *p* < 0.05 was considered statistically significant.

## 5. Conclusions

Fibrin-PRGF membranes improved chemically induced lesions in a rabbit alkali-burn model but only fibrin-PRGF membranes with autologous or heterologous cultured rabbit LEPCs were able to restore the corneal surface. SLET technique with a fibrin-PRGF membrane did not show satisfactory results in this study. Additional data should be collected with a larger sample size, at a longer follow-up and with a different experimental design.

## Figures and Tables

**Table 1:** Clinical evaluation of the rabbits the day of the evaluation and treatment. S0–S4: scores defined in Table 4.

		Corneal Opacification					Corneal Neovascularization					Fibrovascular Pannus					Epithelial Defects				
	Rabbits	S0	S1	S2	S3	S4	S0	S1	S2	S3	S4	S0	S1	S2	S3	S4	S0	S1	S2	S3	S4
**Group 1**	1					●				●				●						●	
	2				●				●					●					●		
	3			●					●				●					●			
**Group 2**	4				●					●					●			●			
	5					●					●					●				●	
	6				●						●				●				●		
**Group 3**	7				●					●					●			●			
	8			●							●					●				●	
	9				●						●				●					●	
**Group 4**	10				●						●			●				●			
	11			●						●				●					●		
	12				●						●					●			●		
**Group 5**	13				●						●				●					●	
	14				●						●				●					●	
	15			●						●					●				●		

Group 1: only medical treatment (control group), Group 2: fibrin-PRGF membrane cultured with autologous rabbit LEPCs, Group 3: fibrin-PRGF membrane cultured with heterologous rabbit LEPCs, Group 4: fibrin-PRGF membrane SLET and Group 5: fibrin-PRGF membrane without rabbit cultured LEPCs. PRGF: Plasma Rich in Growth Factors, LEPCs: Limbal Epithelial Progenitor Cells, SLET: Simple Limbal Epithelial Transplantation.

**Table 2:** Clinical evaluation of the rabbits on the day of the diagnosis and treatment and at the end of the follow-up period. S0–S4: scores defined in Table 4.

		Corneal Opacification					Corneal Neovascularization					Fibrovascular Pannus					Epithelial Defects				
	Rabbits	S0	S1	S2	S3	S4	S0	S1	S2	S3	S4	S0	S1	S2	S3	S4	S0	S1	S2	S3	S4
**Group 1**	1					●/◊				●	◊			●	◊					●	◊
	2			◊	●				●/◊					●/◊				◊	●		
	3			●/◊					●/◊				●/◊					●/◊			
**Group 2**	4	◊			●			◊		●		◊			●		◊	●			
	5			◊		●			◊		●		◊			●	◊			●	
	6	◊			●			◊			●		◊		●			◊	●		
**Group 3**	7	◊			●			◊		●		◊			●		◊	●			
	8	◊		●				◊			●		◊			●	◊			●	
	9	◊			●				◊		●		◊		●			◊		●	
**Group 4**	10	◊			●				◊		●	◊		●			◊	●			
	11			●/◊						●/◊			◊	●				◊	●		
	12	◊			●					◊	●			◊		●		◊	●		
**Group 5**	13		◊		●					◊	●		◊		●			◊		●	
	14		◊		●				◊		●			◊	●		◊			●	
	15		◊	●						●/◊				◊	●			◊	●		

●: pre-treatment; ◊: post-treatment. Group 1: only medical treatment (control group), Group 2: fibrin-PRGF membrane cultured with autologous rabbit LEPCs, Group 3: fibrin-PRGF membrane cultured with heterologous rabbit LEPCs, Group 4: fibrin-PRGF membrane SLET and Group 5: fibrin-PRGF membrane without rabbit cultured LEPCs. PRGF: Plasma Rich in Growth Factors; LEPCs: Limbal Epithelial Progenitor Cells; SLET: Simple Limbal Epithelial Transplantation.

**Table 3:** Percentage of change before and after treatment for each clinical variable measured. Data are shown as mean ± standard deviation (SD).

	Corneal Opacification	Corneal Neovascularization	Fibrovascular Pannus	Epithelial Defects
**Group 1**	−11.11 ± 19.25	11.11 ± 19.25	16.67 ± 28.87	−5.56 ± 41.94
**Group 2**	−83.33 ± 28.87	−63.89 ± 12.73 ^†^	−80.56 ± 17.35 ^†^	−83.33 ± 28.87
**Group 3**	−100.00 ± 00.00	−63.89 ± 12.73 ^‡^	−80.56 ± 17.35 ^‡^	−88.89 ± 19.25
**Group 4**	−66.67 ± 57.74	−25.00 ± 25.00	−66.67 ± 28.87	−66.67 ± 28.87
**Group 5**	−61.11 ± 9.62	−25.00 ± 25.00	−44.44 ± 19.25	−72.22 ± 25.46
***p*** **-value**	0.079	0.032 *	0.040 *	0.126

* Statistically significant difference (*p* < 0.05) between groups. † and ‡, statistically significant difference (*p* < 0.05) between rabbits treated with fibrin-PRGF membrane cultured with autologous (group 2) or heterologous (group 3) rabbit LEPCs and control group. Group 1: only medical treatment (control group), Group 2: fibrin-PRGF membrane cultured with autologous rabbit LEPCs, Group 3: fibrin-PRGF membrane cultured with heterologous rabbit LEPCs, Group 4: fibrin-PRGF membrane SLET and Group 5: fibrin-PRGF membrane without rabbit cultured LEPCs. PRGF: Plasma Rich in Growth Factors; LEPCs: Limbal Epithelial Progenitor Cells; SLET: Simple Limbal Epithelial Transplantation.

**Table 4:** Scores for clinical evaluation.

	Score 0	Score 1	Score 2	Score 3	Score 4
**Corneal Opacification**	Totally clear	Haze of minimal density	Mild haze	Moderately dense opacity	Severely dense opacity
**Corneal Neovascularization**	No vessels	<1/4	>1/4 and <1/2	>1/2 and <3/4	>3/4
**Fibrovascular Pannus**	0	1	2	3	>4
**Epithelial Defects**	No fluorescein stain	<1/4	>1/4 and <1/2	>1/2 and <3/4	>3/4

## Data Availability

All the obtained data used to support the findings of this study are available from the corresponding author upon reasonable request.

## Abbreviations

PRGF	Plasma Rich in Growth Factors
LEPCs	Limbal Epithelial Progenitor Cells
SLET	Simple Limbal Epithelial Transplantation
LSCD	Limbal Stem Cell Deficiency
DMEM	Dulbecco’s modified Eagle’s medium
FBS	fetal bovine serum
CK	cytokeratin
PBS	phosphate buffered saline
DAPI	4′,6-diamidino-2-phenylindole
EMA	European Medicines Agency
ATMP	Advanced Therapy Medicinal Product
